# Increased Prevalence of Anellovirus in Pediatric Patients with Fever

**DOI:** 10.1371/journal.pone.0050937

**Published:** 2012-11-30

**Authors:** Erin McElvania TeKippe, Kristine M. Wylie, Elena Deych, Erica Sodergren, George Weinstock, Gregory A. Storch

**Affiliations:** 1 Department of Pediatrics, Washington University School of Medicine, Saint Louis, Missouri, United States of America; 2 The Genome Institute, Washington University School of Medicine, Saint Louis, Missouri, United States of America; 3 Department of Medicine, Washington University School of Medicine, Saint Louis, Missouri, United States of America; University of North Carolina School of Medicine, United States of America

## Abstract

The *Anelloviridae* family consists of non-enveloped, circular, single-stranded DNA viruses. Three genera of anellovirus are known to infect humans, named TTV, TTMDV, and TTMV. Although anelloviruses were initially thought to cause non-A-G viral hepatitis, continued research has shown no definitive associations between anellovirus and human disease to date. Using high-throughput sequencing, we investigated the association between anelloviruses and fever in pediatric patients 2–36 months of age. We determined that although anelloviruses were present in a large number of specimens from both febrile and afebrile patients, they were more prevalent in the plasma and nasopharyngeal (NP) specimens of febrile patients compared to afebrile controls. Using PCR to detect each of the three species of anellovirus that infect humans, we found that anellovirus species TTV and TTMDV were more prevalent in the plasma and NP specimens of febrile patients compared to afebrile controls. This was not the case for species TTMV which was found in similar percentages of febrile and afebrile patient specimens. Analysis of patient age showed that the percentage of plasma and NP specimens containing anellovirus increased with age until patients were 19–24 months of age, after which the percentage of anellovirus positive patient specimens dropped. This trend was striking for TTV and TTMDV and very modest for TTMV in both plasma and NP specimens. Finally, as the temperature of febrile patients increased, so too did the frequency of TTV and TTMDV detection. Again, TTMV was equally present in both febrile and afebrile patient specimens. Taken together these data indicate that the human anellovirus species TTV and TTMDV are associated with fever in children, while the highly related human anellovirus TTMV has no association with fever.

## Introduction

In 1997 the novel virus Torque Teno Virus (TTV) was isolated from a Japanese patient who developed post-transfusion non-A-G hepatitis [1]. Further analysis classified TTV as a non-enveloped, single-stranded DNA virus with a circular, negative-sense genome [1], [2]. In 2000, researchers using TTV-specific PCR primers amplified a smaller virus which was subsequently named Torque Teno-like Mini Virus (TTMV) [3]. A third virus with a genomic size in between that of TTV and TTMV was discovered in 2007 and subsequently named Torque Teno-like Midi Virus (TTMDV) [4]. Recent changes in nomenclature have classified the three anelloviruses able to infect humans into Alphatorquevirus (TTV), Betatorquevirus (TTMV), and Gammatorquevirus (TTMDV) Genera of the Anelloviridae family of viruses [5]. Despite the early association with hepatitis, further studies have ruled out anelloviruses as a cause of clinically significant liver disease [6], [7], [8]. Researchers have studied anelloviruses in the context of many diseases. Associations between anellovirus and acute respiratory disease have been described, but it is difficult to determine causation vs. correlation due to the ubiquitous nature of anelloviruses [9], [10], [11]. To date anelloviruses are still considered “orphan” viruses waiting to be linked to human disease [12].

As indicated by their names, the human anelloviruses differ in genome size ranging from 3.8–3.9 kb for TTV, 3.2 kb for TTMDV, and 2.8–2.9 kb for TTMV. A characteristic feature of anelloviruses is the extreme diversity found both within and between anellovirus species; they can exhibit as much as 33%–50% divergence at the nucleotide level [3], [13], [14], [15], [16], [17], [18], [19], [20]. Despite the nucleotide sequence diversity, anelloviruses share conserved genomic organization, transcriptional profiles, a non-coding GC rich region, and sequence motifs resulting in shared virion structure and gene functions [3], [21], [22], [23], [24], [25], [26], [27].

Anellovirus infections are highly prevalent in the general population. A study in Japan found that 75–100% of patients tested were infected with at least one of the three human anelloviruses, and many were infected with multiple species [28]. Anelloviruses can infect young children, with the earliest documented infections occurring within the first months of life [29]. These viruses have been found in nearly every body site, fluid, and tissue tested including blood plasma, serum, peripheral blood mononuclear cells (PBMCs), nasopharyngeal aspirates, bone marrow, saliva, breast milk, feces, as well as various tissues including thyroid gland, lymph node, lung, liver, spleen, pancreas, and kidney [2], [25], [30], [31], [32], [33], [34], [35], [36]. The replication dynamics of anelloviruses are virtually unknown because of the inability to propagate these viruses in culture. Positive-strand TTV DNA, indicative of local viral replication, has been described in hepatocytes, bone marrow cells, and circulating PBMCs [37], [38], [39]. Another report proposes that anelloviruses replicate exclusively in tissue because the viral load can be up to 300 times higher than that detected in plasma. This explanation is complicated due to the highly variable viral loads detected in tissue and plasma from patient to patient. More research is necessary to determine the exact site of anellovirus replication [36].

Antibody detection of anellovirus infection has been reported by several labs, but usage of these assays has not spread beyond the originating laboratories. The largest study by Ott *et al*.–consisting of 70 adults and children–identified TTV antibodies in 98.6 percent of patients tested. TTV-IgM antibodies were negative for all of the patients suggesting that the TTV infections were not newly acquired. TTV DNA was detected in 76.1% of the sera, indicating active infection [40]. High viral titers found in patient specimens suggest that anelloviruses cause chronic, persistent infection for life.

Anelloviruses are spread primarily through fecal-oral transmission, although mother-child and respiratory tract transmissions have also been reported [9]; [2], [41]. There are conflicting reports regarding the presence of TTV in cord blood specimens [42], [43]. Analysis of TTV DNA from infected mothers and infants showed that the strains were highly related in only a small number of cases, suggesting that vertical transmission is responsible for a very small amount of anellovirus infections [44].

Fever is one of the most common reasons children go to the emergency department, resulting in over 5.6 million visits in 2007 [45]. In the past, bacteremia was a common source of fever in children. The recent development of *Haemophilus influenzae* and *Streptococcus pneumoniae* vaccines have reduced the frequency of serious bacterial infections in children, making bacteremia an uncommon cause of fever. Localized bacterial infection, such as urinary tract infections, cause 5–10% of fevers in children, but there remains a large percentage of febrile children in which the source of their fever is never identified. Viral illnesses are believed to cause the majority of cases of fever without a source and result in significant morbidity and mortality especially in young children [46]. Viruses such as human herpes virus 6, Epstein–Barr virus, and adenovirus are known to cause fever in children [47], [48], [49], and advances in molecular diagnostic techniques have led to the discovery of unknown or underappreciated viruses associated with human disease. In a study of fever in children, we commonly found anelloviruses in our specimens based on high-throughput sequencing [50]. Therefore we were interested in further investigating the association between anelloviruses and fever in children.

## Results

### Anelloviruses were more Prevalent in Febrile Patient Specimens by High-throughput Sequencing

**Figure 1:**
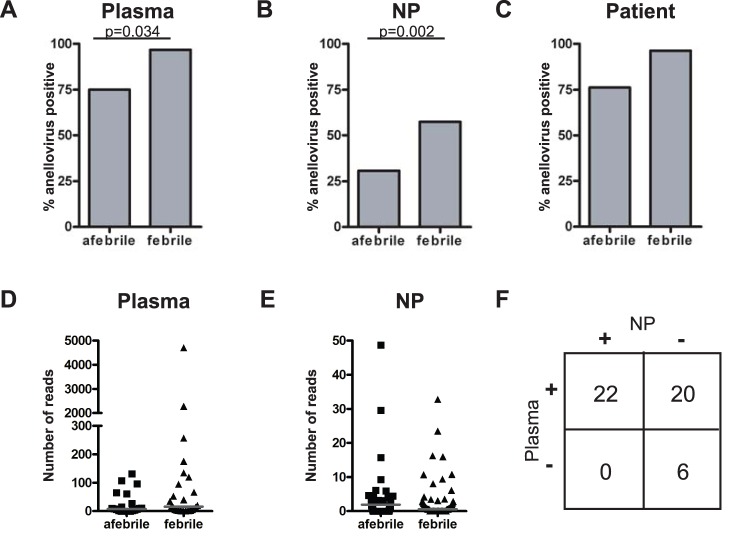
Anellovirus DNA was more prevalent in specimens from febrile children by high-throughput sequencing. **A–B**. High-throughput sequencing analysis of plasma (**A**) and NP (**B**) specimens showed that a higher percentage of specimens from febrile children contained anellovirus DNA compared to specimens from afebrile children (plasma, p = 0.034; NP, p = 0.002). **C**. The trend was the same, but not statistically significant at the patient level of analysis. **D–E**. Afebrile and febrile patients had a similar median number of anellovirus sequence reads in their plasma (10.2 vs. 15.6) (**D**) and NP (2.9 vs. 1.7) (**E**) specimens. **F**. Anellovirus sequences detected by high-throughput sequencing were more prevalent in plasma compared to NP specimens (p<0.0001; Chi-squared test).

To identify viruses associated with fever in children we performed high-throughput sequencing on 52 plasma and 151 NP specimens using the Illumina GA IIx platform. These specimens were obtained from children 2–36 months of age presenting to the Emergency Department at Saint Louis Children’s Hospital with a temperature greater than or equal to 38 degrees Celsius. Afebrile controls consisted of children in the same age range who were having ambulatory surgery for non-acute conditions. High-throughput sequencing was chosen because it is an unbiased approach that can lead to identification of known viruses and has the possibility of uncovering novel viruses as well [50]. After sequencing we found that many of the specimens from our patient cohort contained anellovirus DNA. Anellovirus DNA was present in plasma and NP specimens from both febrile and afebrile patients. In each case further analysis showed that a higher percentage of specimens from febrile patients contained anellovirus DNA compared to specimens from afebrile patients (plasma, p = 0.034; NP p = 0.002) (**Fig. 1A–B**). Patient-level analysis showed that patients with fever were more likely to have anelloviruses detected in one or both samples than were afebrile patients, although the data did not reach statistical significance (**Fig. 1C**). Overall these data suggest an association between children being febrile and having anellovirus DNA detectable in their plasma and NP specimens.

Although high-throughput sequencing is not precisely quantitative, we hypothesized that a larger number of anellovirus reads could be suggestive of a higher viral load. In order to compare samples, the total number of reads was normalized to 3 million reads per specimen. Plasma specimens from afebrile patients contained a similar number of anellovirus sequence reads compared to those from febrile patients, with a median of 10.2 and 15.6 reads, respectively (**Fig. 1D**). The median number of anellovirus sequence reads was also similar between afebrile and febrile NP specimens at 2.9 and 1.7 reads, respectively (**Fig. 1E**). The range of anellovirus sequence reads was higher in plasma specimens, ranging from one to nearly five thousand reads per specimen. Anellovirus-positive NP specimens all had fifty or fewer sequence reads per specimen. These data suggest that the anellovirus viral load is likely similar between febrile and afebrile patients and that the amount of anellovirus present in plasma specimens varies widely between individuals.

To determine if anellovirus DNA was more likely to be found in the plasma or NP specimens of our patient cohort we analyzed 48 patients who had high-throughput sequencing performed on both their plasma and NP specimens. Anellovirus DNA was detected in the plasma and NP specimens of 22 patients and detected in neither specimen for 6 patients (p = 0.0001) (**Fig. 1F**). Twenty patients had anellovirus sequences detected in their plasma but not NP specimens. These data suggest that the anellovirus viral load is below the level of detection in the upper respiratory tract or that many children develop systemic anellovirus infection that involves the blood but not the upper respiratory tract.

### Human Anellovirus Species TTV and TTMDV were more Prevalent in Febrile Patient Specimens by PCR

**Figure 2:**
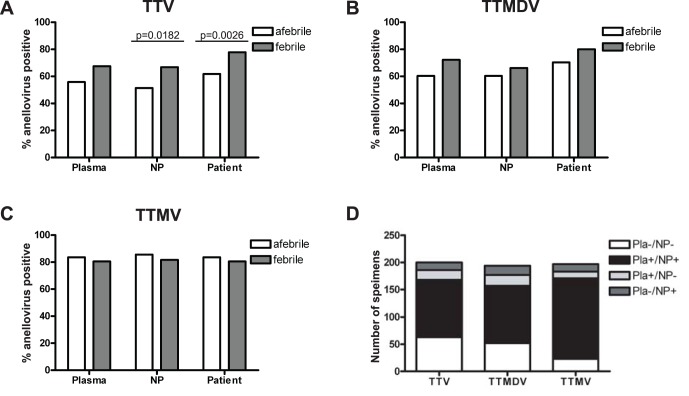
PCR analysis showed that DNA from human anellovirus species TTV and TTMDV, but not TTMV, were more prevalent in febrile patient specimens compared to afebrile controls. **A–C**. PCR identified TTV (**A**) and TTMDV (**B**) in a higher percentage of febrile patients compared to afebrile controls while TTMV (**C**) was present in equivalent percentages (plasma TTV, p = 0.0567; NP TTV, p = 0.0182; patient TTV, p = 0.0026). **D**. The majority of patients were either positive or negative for TTV, TTMDV, and TTMV DNA in both their plasma and NP specimens. P-values determined by chi-squared test.

After making our initial observations using high-throughput sequence data, we expanded our analysis to a larger set of specimens using PCR assays designed to detect TTV, TTMDV, and TTMV**.** We hypothesized that specific anellovirus species were associated with fever in children while other species may have no association. To test our hypothesis we developed conventional PCR assays to detect TTV, TTMDV, and TTMV using primers validated by Ninomiya *et al.* [29]. PCR for TTV, TTMDV, and TTMV was performed on 219, 213, 216 plasma specimens, respectively. All three PCR assays were also performed on 247 NP specimens. Patient-level analysis was performed to evaluate individuals with anellovirus DNA in their plasma and/or NP specimens. In agreement with high-throughput sequencing, anellovirus DNA was detected in a high percentage of specimens from both febrile and afebrile patients. Analysis of the two specimen types for specific anellovirus species showed that TTV DNA was present in a higher percentage of NP specimens from febrile patients compared to afebrile controls (p = 0.0182); results were similar, but not statistically significant, for plasma specimens (**Fig. 2A**). The same trend was true for TTMDV, but to a lesser extent than TTV (**Fig. 2B** **)**. These results indicate an association between infection with TTV and possibly TTMDV and fever in children. In contrast, TTMV DNA was found in similar percentages of febrile and afebrile patient specimens indicating that TTMV infection is not associated with fever in children (**Fig. 2C** **)**. Patient level analysis was performed in which patients with TTV, TTMDV, or TTMV DNA in their plasma, NP, or both specimens were considered positive. This analysis showed that approximately 80% of all patients were positive for anellovirus regardless of their febrile or afebrile status, and that TTV was present in a higher percentage of febrile patients compared to afebrile controls (TTV, p = 0.0026). TTMDV followed the same trend as TTV, but the difference between febrile patients and afebrile controls did not reach statistical significance. The majority of patients in our cohort were positive for TTV, TTMDV, or TTMV DNA in both their plasma and NP specimens (**Fig. 2D**). The next largest group consisted of patients without TTV, TTMDV, or TTMV DNA detected in either their plasma or NP specimens. Depending on the species, only 13–19 percent of patients were positive for TTV, TTMDV, or TTMV DNA in only one specimen. The frequency was equally split between patients positive in only their plasma or NP specimen.

Our data show that TTV and TTMDV, but not TTMV, are associated with fever in children. These data indicate that the three human anellovirus species are not interchangeable, and suggest that TTV, TTMDV, and TTMV play different roles in pathogenesis and disease.

### Patient Specimens Contain Multiple Human Anellovirus Species

**Figure 3:**
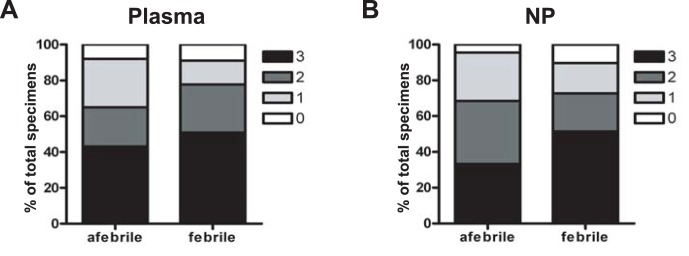
Many patient specimens contained DNA from multiple human anellovirus species. **A–B**. Patient plasma (**A**) and NP (**B**) specimens were assayed for TTV, TTMDV, and TTV DNA by PCR and the percentage of specimens with 3, 2, 1, or no anellovirus species were determined.

To determine if patients in our cohort were co-infected with multiple anellovirus species, we analyzed our PCR data for the simultaneous presence of TTV, TTMDV, and TTMV DNA in plasma and NP specimens. We found that the majority of specimens were positive for more than one human anellovirus species (**Fig. 3A–B**). This was true of specimens from both febrile and afebrile patients. The largest group consisted of specimens containing all three anellovirus species while less than ten percent of specimens were negative for all three human anelloviruses.

To investigate whether anelloviruses formed synergistic relationships with other known viral pathogens we analyzed the presence of anelloviruses with respect to 20 other common viruses that were previously tested for in the same samples (ref Colvin). (data not shown). Our analysis showed that specimens with anelloviruses were not more likely to be positive for other viruses than specimens without anelloviruses. This was true for the individual viruses we tested, as well as for viruses as a whole. Thus, we did not find any associations between TTV, TTMDV, or TTMV and other viral pathogens, suggesting that anelloviruses do not have synergistic interactions with other common viral pathogens.

### Associations with Age, Gender, and Race

**Figure 4:**
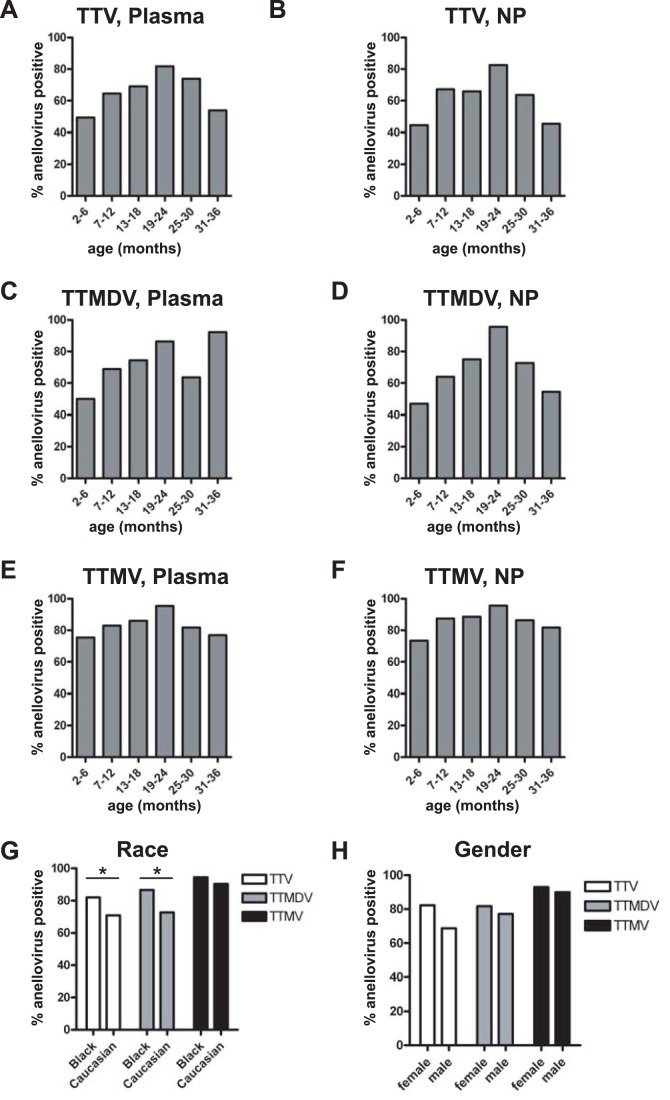
Anellovirus association with patient age, race, and gender. **A–F**. Children in whom anellovirus DNA was detected were older than children in whom anellovirus DNAS was not detected. Patients were broken down by age into 6 month age groups and analyzed for the presence of DNA from TTV (**A–B**), TTMDV (**C–D**), or TTMV (**E–F**) in patient plasma (**A, C, E**) or NP (**B, D, F**) specimens. **G**. Other demographic analysis showed that anellovirus species TTV and TTMDV were more prevalent in patient specimens from African-Americans than Caucasians, but using multivariate logistic models the race effect was not significant after adjusting for the febrile status. **H**. There was no correlation between anellovirus positivity and patient gender.

To investigate the relationship between anelloviruses and patient demographics we used our PCR data to assess the prevalence of anellovirus in relation to patient age, race, and gender. Patients infected with TTV and TTMDV had a higher median age than uninfected patients (median age TTV pos = 12 months vs. TTV neg = 8 months (p = 0.015 by Kolmogorov-Smirnov test); TTMDV pos = 12 months vs. TTMDV neg = 6 months (p = 0.002, Kolmogorov-Smirnov test); data not shown). Children infected with the anellovirus species TTMV had comparable mean and median ages to children who were uninfected (TTMV pos = 11 months vs. TTMV neg = 12 months; data not shown). It is logical that patients are exposed to more viruses as they age, but the similar median age of TTMV positive and negative children demonstrates that the age effect we see with TTV and TTMDV is not due solely to patients acquiring common viruses as they age. To investigate the association between anelloviruses and age more closely we combined patients into groups consisting of six month age blocks. We then analyzed the percentage of children in each age group who were positive for TTV, TTMDV, and TTMV DNA in plasma or NP specimen. The youngest group, consisting of children 2–6 months of age, had relatively low percentages of TTV and TTMDV DNA in their plasma and NP specimens (**Fig. 4A–D**). The percentage of anellovirus positive specimens climbed with increasing age, peaking at 19–24 months, and then declined from 25–36 months. A very modest rise and fall was seen in TTMV-positive plasma and NP specimens (**Fig. 4E–F**). We expected to find a lower frequency of anellovirus infections in younger patients, but were surprised to see the frequency of anellovirus peak and then drop after 24 months of age. These data suggest that a subset of patients may be able to clear anelloviruses from their bodies or suppress anellovirus replication to below the level of detection for our PCR assay.

Another parameter we investigated was the race of patients infected with anelloviruses. We found that DNA from TTV and TTMDV, but not TTMV, was more prevalent in African-American patients compared to Caucasian patients (**Fig. 4G**). Our analysis was complicated by the fact that more African-American patients were in our febrile group compared to our afebrile control group due to the usage patterns of the Emergency Department and Ambulatory Surgery. Conversely, Caucasian patients were over represented in our afebrile control group. In multivariate logistic models, race was not significantly assoicated with the presence of anelloviruses after adjusting for the febrile status. Patients classified as Asian, Hispanic, and other were present in such small numbers that they were excluded from this analysis. We also analyzed the presence of anelloviruses in male and female patients, and found no significant differences according to gender (**Fig. 4H**).

### Anellovirus Infection Correlates with Elevated Temperatures in Children

**Figure 5:**
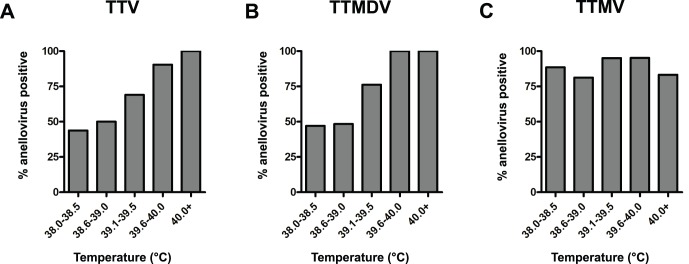
Detection of anellovirus DNA correlates with increasingly elevated temperatures in children. **A–B**. The percent of patients infected with detectable TTV (**A**) and TTMDV (**B**) rises as patient's temperature increases. **C**. The percentage of patients with TTMV DNA detected is independent of temperature.

The maximum temperature of all patients in our cohort was recorded and any patient with a temperature of 38°C or higher was considered febrile for the purposes of our study. Children were then grouped by one-half degree Celsius increments in their maximum temperatures, and we determined the percent of children in each group who were positive for TTV, TTMDV, or TTMV DNA. There was a striking correlation between the height of the maximum temperature and the frequency of TTV and TTMDV infection (**Fig. 5A–B**). Fewer than half of children with maximum temperatures of 38–38.5°C were positive for TTV or TTMDV DNA. In contrast, nearly 100% of children with a maximum temperature of 40°C or higher were infected with TTV or TTMDV. The presence of TTMV DNA did not have a similar correlation with maximum temperature as TTV and TTMDV (**Fig. 5** **C**). Regardless of the height of the maximum temperature, 80–90% of febrile children were infected with TTMV.

## Discussion

The focus of our research study was to investigate the association of individual anellovirus species with febrile illness in young children. Interestingly, we found that TTV, TTMDV, and TTMV do not exhibit the same biological behavior. A paper by Rocchi *et al.* speculated that the sequence diversity found within and between anellovirus species may affect the immune response to the virus, resulting in different effects on the body during infection [51]. In our study TTV and TTMDV were associated with febrile illness. In contrast, TTMV was equally common in febrile and afebrile children.

Early research on anelloviruses drastically underreported their prevalence in patient specimens. The *Anellovirdae* family of viruses is extremely diverse. They have few conserved regions between species and also within species, making it difficult to design PCR primers that amplify all anelloviruses that might be present in patient specimens [52], [53]. Continued research has revealed a high level of diversity of anellovirus at the nucleotide level, and appreciation of this diversity has led to higher frequency of detection of anelloviruses in patient specimens, including healthy controls [12], [29]. We used conventional end point PCR assays to detect TTV, TTMDV, and TTMV. To ensure that our PCR assays were achieving maximum broad range detection of individual anellovirus species (TTV, TTMDV, and TTMV) as possible, we used primers that were homologous to sequences within the GC rich region upstream of the OFR1 transcription start site. This is the most conserved region in the *Anellovirdae* family of viruses but also provides conserved sequences that are unique for each species of viruses. We used multiple forward and reverse primers to maximize the range of detection within each anellovirus species. Synthetic oligonucleotides representing two diverse sequences within each anellovirus species were used as positive controls to ensure that our PCR primers and conditions were suitable for amplification of diverse anellovirus sequences.

In our study we investigated various patient demographic parameters to determine whether they correlated with the presence of anelloviruses in our patient cohort. We were also interested in racial differences in virus distribution. Using univariate analyses, African-American subjects had a significantly higher prevalence of anelloviruses. However, in our population race was highly correlated with febrile status–57% of febrile children were African-American and 43% were Caucasian, while 15% of afebrile controls were African-American and 85% were Caucasian. In multivariate logistic models race was not significant after adjusting for the febrile status. Since race and fever status were so highly collinear, we assessed the effect of race in analyses stratified by maximum temperature. In a subsample of afebrile subjects African-American subjects had significantly higher presence of TTV virus than Caucasian subjects (OR: 4.9, 1.01–23.35, p = 0.048). No racial differences were found in febrile patients for TTV or for either febrile or afebrile for TTMDV.

We also investigated the age of patients infected with anellovirus. Based on the concept that anelloviruses cause persistent infection, we hypothesized that older children would be more likely than younger children to be infected due to a longer exposure time to anellovirus-positive family members and other children. We found that children with TTV and TTMDV DNA detected in their plasma or NP specimens were older than uninfected children. This was not the case for TTMV as children in which TTMV DNA was detected were no different in age from those in which TTMV DNA was not detected. After grouping our patient cohort into 6 month age blocks we found that the percentage of anellovirus-positive specimens rose as age increased and peaked at 19–24 months, after which the percent of positive specimens declined. The trend was striking in the TTV and TTMDV-positive plasma and NP specimens and modest in the TTMV-positive plasma and NP specimens. This was a surprising finding because research in the anellovirus field has shown that they are chronic, replicating viruses, with no evidence that they are cleared from body or enter into a latent life cycle phase. We saw the same trend in plasma and NP specimens tested for all three human anellovirus species suggests that the finding is real and that further analysis is needed to explain this phenomenon. In our study, each patient was sampled only once; however, longitudinal studies in which the onset of anellovirus infection and the viral load could be assessed would allow us to address the question of anellovirus persistence.

Our study showed that DNA from anellovirus species TTV and TTMDV, but not TTMV, were related to fever in young children. Most strikingly we found that the higher the temperature, the larger the percentage of patients that were positive for TTV and TTMDV. Although the relationship is clear, the mechanism by which this happens is unknown. TTV and TTMDV could be causing fevers in children, but we believe it is more likely that fever brought on by other means is creating a permissive environment for anellovirus replication or decreasing the clearance of the viruses. The combination of an inflammatory environment and a weakened immune system could alter the replication and degradation dynamics affecting infection, proliferation, and stability of TTV and TTMDV. To address this question one would need to follow a cohort of patients over time using a quantitative assay to measure viral titers of TTV and TTMDV. This type of experimentation would allow one to study the effect of a patient’s first anellovirus infection as well as anellovirus replication dynamics as patients undergo various other febrile illnesses. It would also be interesting to follow anellovirus titers before and after patients have been placed on immunosuppressant drug regimens to study the role of the immune system in anellovirus replication.

In conclusion, our study investigated the interplay between anelloviruses and fever. By high-throughput sequencing we found more anellovirus DNA in the plasma and NP specimens of febrile patients compared to afebrile controls. PCR detection of each anellovirus species showed that TTV and TTMDV, but not TTMV, were more prevalent in febrile patient specimens compared to afebrile controls. Finally, as the temperature of febrile patients increased, so too did the frequency of detection of DNA from TTV and TTMDV. Overall these data argue that the anelloviruses TTV and TTMDV are associated with fever in children.

## Materials and Methods

### Patient Population

Febrile children age 2–36 months were recruited from the Emergency Department and afebrile controls were recruited from the Ambulatory Surgery Department at St. Louis Children's Hospital. Febrile children consisted of children with a temperature greater than 38°C without an apparent source, who were having blood obtained for a complete blood count and/or a blood culture for clinical management. Children with clinical syndromes suggestive of viral respiratory infection, such as bronchiolitis, were not included. The elevated temperature was documented either in the Emergency Department or by a health care provider within 24 hours before the Emergency Department evaluation. Children were excluded if they had an underlying condition that predisposed them to infection, including cancer, immune deficiency, immunosuppressive therapy, cystic fibrosis, sickle-cell disease, or presence of an indwelling venous catheter. Children with a positive rapid test for influenza also excluded. The afebrile control group consisted of well children 2–36 months of age having outpatient surgery who had been afebrile for at least 7 days prior to surgery. Enrollment Children with fever were enrolled from mid-February, 2007 through mid-February, 2010. Afebrile control children were enrolled during a 12-month period starting in mid-February 2009. Children were enrolled by study personnel who obtained health information from each child’s caregiver, including past history and recent symptoms that might indicate the presence of an acute infection [54].

### Patient Specimens

Plasma and nasopharyngeal swabs were obtained from febrile and afebrile patients and analyzed for the presence of anellovirus DNA by high-throughput sequencing and PCR. No patient had more than one specimen from either body site. The decision to obtain blood and nasopharyngeal specimens was made by Emergency Department physicians as part of their standard care and was not a part of this study.

### Ethics Statement

This study was initially approved by the Washington University Human Research Protection Office and undergoes yearly review. Written informed consent was obtained from appropriate caregivers.

### High-throughput Sequencing

52 plasma and 151 NP specimens underwent high-throughput sequencing using the Illumina GA IIx platform to determine the presence or absence of anellovirus DNA in plasma and NP patient specimens. Illumina deep sequencing produced read lengths of 100 bp each, and a mean of 4.7 million reads per specimen. To achieve consistent comparisons between groups, the number of reads was scaled to three million reads per specimen. The details of the sequence analysis along with the mean, range, and median number of reads for this data set can be found in figures S1 and S5 of the manuscript published by Wylie *et al.* in PLoS ONE (2012) [50].

### PCR Assays

Plasma and NP specimens were analyzed for the presence or absence of TTV, TTMDV, and TTMV DNA by conventional PCR. We tested 247 NP specimens for all three human anelloviruses. Of the plasma specimens, we tested 219, 213, and 216 for TTV, TTMDV, and TTMV, respectively, due to limited amounts of specimen for some patients. Individual PCR reactions were developed for each of the three human anelloviruses. Conditions for our TTV specific PCR assay included an initial denaturation phase of 2 min. at 94°C followed by 40 cycles of amplification (94°C for 30 sec, 55°C for 30 sec, 72°C for 30 sec), and an extension phase of 7 min. at 72°C. TTMDV conditions were similar with exception of 45 cycles of amplification and an annealing temperature of 60°C. TTMV required a hemi-nested PCR. The first round required an annealing temperature of 60°C and 25 cycles of amplification. The second round required an annealing temperature of 55°C and 25 cycles of amplification. Two µl of extracted nucleic acid from our clinical specimen were amplified by PCR using multiple forward and reverse primers adapted from Ninomiya *et al*. [29]: TTV (RGTGRCGAATGGYWGAGTTT, ACWKMCGAATGGCTGAGTTT, CCCCTTGACTBCGGTGTGTAA); TTMDV (GCCCGARTTGCCCCTAGACC, SGABCGAGCGCAGCGAGGAG); TTMV round one (RGTGRCGAATGGYWGAGTTT, ACWKMCGAATGGCTGAGTTT, CCCKWGCCCGARTTGCCCCT, AYCTWGCCCGAATTGCCCCT); TTMV round two (TTTATGCYGCYAGACGRAGA, TTTAYCMYGCCAGACGGAGA, TTTATGCCGCCAGACGRAGG, CTCACCTYSGGCWCCCGCCC). Two positive controls from the GC rich region upstream of ORF2, ranging in length from 72–112 base pairs, and consisting of diverse sequences were synthesized for each anellovirus species to ensure our primers were amplifying diversity within each species. The limit of detection was <200 viral copies/ml for all three PCR assays.

### Statistics

Statistical significance was determined by Fisher's exact test (**Fig. 1A–1C**), Kolmogorov-Smirnov test (**1D–E**), McNemar’s test (1F), and chi-squared (**Fig. 2A–C**). Multivariate logistic analysis was performed to determine confounding factors within our data set, adjusting for age, gender, and race (**Fig. 4**). Kolmogorov-Smirnov test was used to compare median age of patients infected with TTV, TTMDV, and TTMV (data not shown). Trends testing was performed on data from figure 5, but it did not reach statistical significance.
